# A Case Report of Systemic Capillary Leak Syndrome: When More Than One Inciting Factor Exists, the Question Is Who Pulls the Trigger?

**DOI:** 10.7759/cureus.77261

**Published:** 2025-01-10

**Authors:** Vasileios Patriarcheas, Eleftheria Ztriva, Vasiliki Gougoula, Michail Makris, Erofili Papathanasiou, Christos Savopoulos, Georgia Kaiafa

**Affiliations:** 1 First Propedeutic Department of Internal Medicine, American Hellenic Educational Progressive Association (AHEPA) University Hospital of Thessaloniki, Aristotle University of Thessaloniki, Thessaloniki, GRC

**Keywords:** clarkson’s disease, covid-19, idiopathic systemic capillary leak syndrome, jak 2 mutation, myeloproliferative neoplasm disease, systemic capillary leak syndrome

## Abstract

Systemic capillary leak syndrome (SCLS) constitutes a rare clinical entity. It is characterized by spontaneous, recurrent episodes of increased capillary permeability, leading to a double clinico-biological paradox: diffuse pitting edema with hypovolemia or hypovolemic shock, and hemoconcentration with hypoalbuminemia, in the absence of secondary causes for such abnormalities. Even though several theories have been proposed, the exact pathophysiology of SCLS remains unclear. We report herein a case of idiopathic SCLS in a 38-year-old male patient with a history of previous immunization, following COVID-19 infection, who was also diagnosed with a myeloproliferative neoplasm (MPN). To our knowledge, this is the first case where SCLS coexists with an MPN, leading to a legitimate question: Who pulls the trigger?

## Introduction

Systemic capillary leak syndrome (SCLS), first described by Clarkson in 1960, constitutes a rare clinical entity. It is characterized by spontaneous, recurrent episodes of increased capillary permeability leading to a double clinico-biological paradox: diffuse pitting edema with hypovolemia or hypovolemic shock and hemoconcentration with hypoalbuminemia, in the absence of secondary causes for such abnormalities. SCLS can be classified as either primary (idiopathic SCLS (ISCLS)) or secondary. While precipitants of acute idiopathic SCLS episodes may not be present, 40-50% of cases are often preceded by infections, predominantly caused by respiratory viral pathogens. On the other hand, secondary SCLS is often attributed to a discernible potential cause, such as drugs-especially antineoplastic and immunomodulatory agents, toxins, environmental exposures, infections, and hematological malignancies such as lymphoma and leukemia [1]. Clinical manifestations of ISCLS include a prodrome of fatigue and flulike symptoms, which is followed by the acute onset of hypovolemic shock and systemic edema due to the extravasation of protein-rich fluid into the interstitial space, leading to a variety of complications, such as acute kidney injury, hepatic failure, ischemic stroke, myocardial infarction, rhabdomyolysis, compartment syndrome, and pleural and pericardial effusions [2,3].

To date, fewer than 350 cases of ISCLS have been reported worldwide, with a mean age of 51 years at diagnosis and no apparent gender predilection [1]. In recent years, there has been a rise in reported cases of ISCLS that were attributed to both COVID-19 infection per se, as well as an adverse event following immunization in previously undiagnosed patients [4,5]. Several hypotheses have been proposed for ISCLS pathophysiology; however, there is insufficient evidence to support any of them. ISCLS is a diagnosis of exclusion and mandates a high index of suspicion. Differential diagnosis is wide and includes septic shock, anaphylaxis, anaphylactic attacks of systemic mastocytosis, hereditary angioedema, drug reactions, and hemophagocytic lymphohistiocytosis [1,2]. We present a diagnostic dilemma of an SCLS case, where more than one factor could serve as a trigger: COVID-19 vaccination, COVID-19 infection, and a myeloproliferative neoplasm (MPN).

## Case presentation

A 38-year-old man, vaccinated against COVID-19 (two doses of BNT162b2 mRNA Pfizer-BioNTech vaccine), with no medical history, presented to the emergency department of a tertiary hospital in July 2022, complaining of a high fever of up to 39°C with general weakness three days prior. He also reported syncope the day before, preceded by diaphoresis and back and abdominal pain. Upon arrival, the patient was hypotensive, tachypneic, and afebrile. The physical examination was unremarkable. Laboratory investigation revealed a positive polymerase chain reaction test for SARS-CoV-2 infection, elevated hemoglobin, acute kidney injury hypoalbuminemia, and moderately elevated C-reactive protein levels (Table 1). Arterial blood gas revealed metabolic acidosis, while computed angiography of the thoracic and abdominal aorta was normal. He was supported with empiric antibiotic therapy, fluid administration, and vasopressors with gradual improvement, and he was discharged eight days later in good condition with restoration of renal function. During the first hospitalization, due to elevated hemoglobin, the patient underwent molecular testing for Janus kinase 2 (JAK2) V617F mutation, which was positive.

**Table 1:** Abnormal patient’s laboratory findings during the first, second, and last admission.

Laboratory findings	First admission	Second admission	Last admission	Normal range
Hemoglobin	24.9 g/dl	23.5 g/dl	19.3 g/dl	14-18 g/dl
Albumin	2.45 g/dl	2.2 g/dl	3.1 mg/dl	3.5-5.1 g/dl
Creatinine	1.96 mg/dl	2.66 mg/dl	1.5 mg/dl	0.8-1.25mg/dl
CRP	1.3 mg/dl	1.5 mg/dl	1.7 mg/dl	<0.5mg/dl
Troponin	12 pg/ml	283 pg/ml	12 pg/ml	<14 pg/ml

Three months later, the patient was admitted to the emergency department of another tertiary hospital due to syncope. He reported symptoms of a common cold in the last 24 hours. At admission, the patient was experiencing severe hypotension and was hemodynamically unstable. Emergency computed tomography of the chest and abdomen was performed without remarkable findings. The initial laboratory investigation showed acute kidney injury, hemoconcentration, hypoalbuminemia, and moderately elevated C-reactive protein levels (Table 1). Due to elevated levels of troponin, the patient was transferred to the Coronary Care Unit, where he was under continuous monitoring. He was supported with fluid resuscitation and administration of vasopressors and albumin. During this hospitalization, clinicians recognized the triad of hypoalbuminemia, hypotension, and hemoconcentration, and they set the diagnosis of SCLS. He was finally discharged, and he was scheduled to receive administration of intravenous immunoglobulin (IVIg) once monthly. Over the next two years, because of poor compliance with scheduled follow-up and IVIg administration, the patient had seven further admissions to several hospitals in Northern Greece, requiring hospitalization due to recurrent episodes of SCLS.

During his last episode, the patient was admitted to our hospital with a similar presentation of symptoms. He was supported with fluid administration and vasopressors, and he also received albumin replacement as well as IVIg, with gradual improvement until he was discharged in good condition, with normalization of laboratory values, and hemodynamically stable. 

Moreover, we decided to proceed to bone marrow biopsy, which confirmed the presence of hypercellular bone marrow and morphological characteristics resembling an MPN.

## Discussion

**Figure 1:**
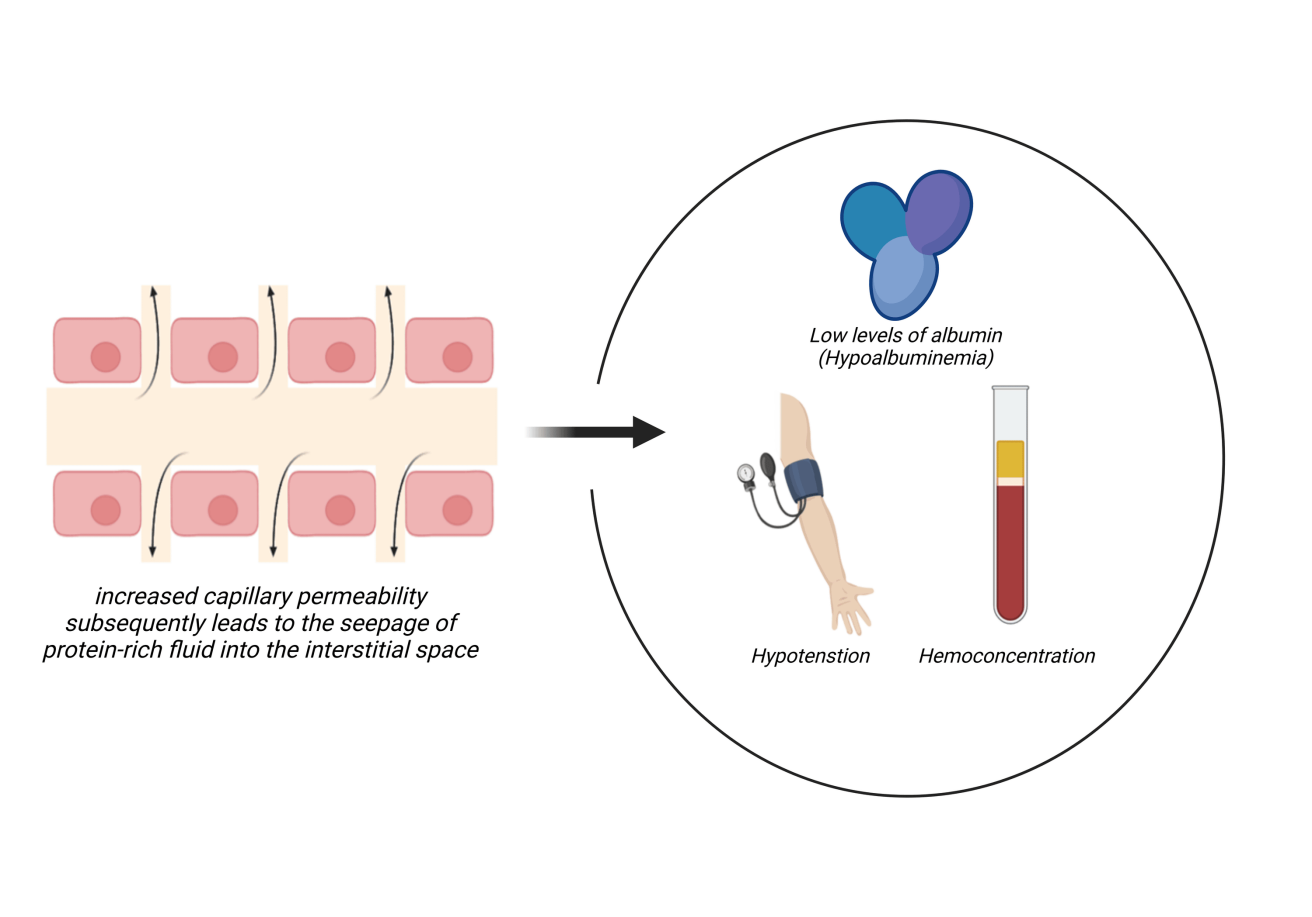
Extravasation of protein-rich plasma to the interstitial space, leads to the 3 “H’s” of ISCLS: hypotension, hemoconcentration and hypoalbuminemia Created in BioRender by Vasileios Patriarcheas (https://BioRender.com/t82m862) ISCLS: Idiopathic systemic capillary leak syndrome

SCLS is an extremely rare and potentially life-threatening entity. During episodes, significant disruptions of the vascular endothelium occur, causing abrupt alterations in capillary permeability that subsequently result in the extravasation of plasma and proteins into the interstitial space leading to the 3H’s: hypotension, hemoconcentration, and hypoalbuminemia (Figure 1).

Acute attacks demonstrate a triphasic pattern consisting of the prodromal, the fluid extravasation, and lastly the recovery phase [6]. In the prodromal phase, there are non-specific symptoms, such as fever, flu-like symptoms, fatigue, generalized weakness, and abdominal pain. The transition to the fluid extravasation phase is abrupt and potentially life-threatening. As this occurs, the patient exhibits severe hypotension, hemoconcentration, and hypoalbuminemia owing to significant plasma extravasation. Finally, the patient enters the recovery phase, where endothelial function is restored, and the extravasated fluids are reabsorbed into the intravascular compartment. In the course of the recovery phase, there is a high risk of pulmonary edema due to iatrogenic fluid overload. The exact pathophysiological mechanism of ISCLS is not well understood, and several hypotheses have been proposed. The most widely accepted theory is that the presence of a soluble component plays a crucial role in the pathogenesis of disease by facilitating the disruption of the endothelial barrier, as it has been demonstrated by in vitro experiments where ISCLS serum-induced permeability in microvascular endothelial cells [7].

The first hypothesis includes monoclonal paraproteins. It is well known that in up to 80% of ISCLS patients, monoclonal gammopathy of undetermined significance (MGUS) is present (mainly IgGk). Binding of paraprotein to the endothelial cells leads to endothelial barrier disruption by F-actin stress fiber formation and VE-cadherin internalization. However, MGUS does not appear to be sufficient to initiate an ISCLS episode, particularly considering that pediatric patients with ISCLS typically do not exhibit MGUS [2,3]. Another hypothesis includes the involvement of interleukin-2 (IL-2), based on the observation that patients treated with high-dose recombinant IL-2 therapy could develop ISCLS. Other theories involve VE-cadherin decrease, as it was observed in animal studies after monoclonal antibody against VE-cadherin (mAbBV13) administration, elevated levels of VEGF-A in ISCLS patients, and their successful outcome after being empirically treated with bevacizumab and inflammatory mediators such as leukotrienes and tumor necrosis factor-alpha (TNF-alpha) [2].

Over the last four years, several case reports have been published highlighting the development of SCLS following COVID-19 infection as well as vaccination, and several theories have been proposed in order to explain the possible causative link with both. Of note in our case, we have three possible mechanisms that could explain the development of SCLS: COVID-19 vaccination, COVID-19 infection, and JAK2 V617F positivity. The emergence of infrequent adverse events following vaccination has gained much clinical scrutiny [8-10]. Activation of CD8+ and CD4+ T-cells, as well as the generation of TNF-α, IFN-γ, and IL-2, appears to be a significant effect of COVID-19 vaccinations. IL-2 promotes the synthesis of nitric oxide synthase (NOS), which facilitates the production of nitric oxide (NO), resulting in capillary damage. Furthermore, TNF-α and IFN-γ can exacerbate capillary leakage due to their direct impact on endothelial cells [4,11]. Our patient had received the BNT162b2 mRNA COVID-19 (Pfizer-BioNTech) vaccine before 10 months, and despite the fact that in the vast majority of cases, SCLS occurs as an adverse event of vaccination, usually after a couple of days or a maximum of a month [3], in one case the time of onset of symptoms after vaccination was one year later [12]. Moreover, our patient during the first episode of SCLS had a mild COVID-19 infection. COVID-19 infection-induced SCLS could be explained as the result of the cytokine storm, which leads to the further activation of immune cells, leading to endothelial injury and enhancement of vascular permeability [13,14].

Finally, prior to the definitive diagnosis of SCLS, the patient underwent additional investigations due to elevated hemoglobin levels and tested positive for the JAK2 mutation. Upon admission to our department for exacerbation of SCLS, a bone marrow biopsy was performed, revealing findings consistent with an MPN. It is well known that JAK2 interacts with endothelial cells and induces the release of neutrophil extracellular traps, which can cause tissue damage and initiate inflammation. This inflammatory state, especially under stress or due to an infection, could lead to endothelial cell damage and capillary leak [15]. The lack of consensus guidelines in combination with the coexistence of an MPN is making this case even more challenging. Given the potential role of the JAK2 mutation in this SCLS case, our concerns remain regarding the management of our patient with a JAK2 inhibitor, as we are uncertain about potential medication-induced exacerbations.

## Conclusions

SCLS is a rare and potentially fatal syndrome, where early recognition and prompt initiation of supportive therapy are prerequisites. In our patient, it is not possible to ascertain which of the aforementioned factors precipitated the development of SCLS. To our knowledge, this is the first case of SCLS coexisting with an MPN.
